# Combining Molecular and Traditional Prognostic Factors: A Holistic Approach to Breast Cancer Prognostication

**DOI:** 10.3390/diagnostics14131449

**Published:** 2024-07-07

**Authors:** Liviu Moraru, Melinda Ildiko Mitranovici, Raluca Moraru, Septimiu Voidazan, Mihai Munteanu, Rares Georgescu, Dan Costachescu, Sabin Gligore Turdean

**Affiliations:** 1 Department of Anatomy, “George Emil Palade” University of Medicine, Pharmacy, Sciences and Technology, 540142 Targu Mures, Romania; liviu.moraru@umfst.ro; 2 Department of Obstetrics and Gynecology, Emergency County Hospital Hunedoara, 14 Victoriei Street, 331057 Hunedoara, Romania; 3 Faculty of Medicine, “George Emil Palade” University of Medicine, Pharmacy, Sciences and Technology, 540142 Targu Mures, Romania; raluca.moraru@umfst.ro; 4 Department of Epidemiology, “George Emil Palade” University of Medicine, Pharmacy, Sciences and Technology, 540142 Targu Mures, Romania; septimiu.voidazan@umfst.ro; 5 Faculty of Electrical Engineering, Technical University, George Baritiu Street, 400394 Cluj Napoca, Romania; mihai.munteanu@ethm.utcluj.ro; 6 Department of Surgery, “George Emil Palade” University of Medicine, Pharmacy, Sciences and Technology, 540142 Targu Mures, Romania; rares1geo@gmail.com; 7 Department of Orthopedisc-Traumatology, Urology, Radiology and Medical Imaging, University of Medicine and Pharmacy Victor Babes, Square Eftimie Murgu, 300041 Timisoara, Romania; costachescu.dan@umft.ro; 8 Department of Pathology, County Clinical Hospital of Targu Mures, 540072 Targu Mures, Romania; sabiturdean@yahoo.com

**Keywords:** breast cancer, prognostic factor, molecular biomarkers

## Abstract

Breast cancer is a heterogeneous disease with various morphologies and molecular features, and it is the second leading cause of cancer death in women in developed countries. According to the literature, we currently lack both prognostic biomarkers and therapeutic targets. The most important prognostic factors are disease stage and Nottingham grade. We conducted a retrospective analysis involving 273 patients with BC who underwent neoadjuvant therapy before proceeding to curative surgical treatment between 1 January 2014 and 31 December 2023. Pathological procedures were conducted at the Department of Pathology, Emergency County Hospital of Targu Mureș, Romania. A statistical analysis was performed. Regarding the relationship between Nottingham grade and Ki67, grade I was associated with a Ki67 of less than 14. The relationship between tumor grade and luminal was similar (*p* = 0.0001): Grade I was associated with luminal A. Regarding TNM stage, it was statistically significantly correlated with TILs (*p* = 0.01) and RCB (*p* = 0.0001). Stages III and IV were associated with a high RCB and poor prognosis. Regarding the prognostic value, Nottingham grade 3 and TNM stages III and IV were correlated with low overall survival and disease-free survival, with poor prognosis, and, among the molecular variables, RCB played the most important prognostic role.

## 1. Introduction

Breast cancer is a heterogeneous disease with various morphologies, molecular features, tumor behaviors, and responses to treatment [1]. Despite early diagnosis and therapeutic advances, breast cancer (BC) is the second leading cause of cancer death in women in developed countries, and it remains a major health problem, accounting for 1.7 million new cancer diagnoses worldwide each year [1,2,3].

The systemic therapy of breast cancer includes chemotherapy, hormonal therapy, and targeted therapy, but NACT (neoadjuvant chemotherapy) is the standard treatment in locally advanced breast cancer [1,2,3,4]. The role of immunotherapy is well known in human epidermal growth factor 2 (HER2)-positive breast cancer patients, but its role in hormone receptor (HR)-positive and HER2-negative BC remains unexplored [5].

Prognostic biomarkers are needed to predict survival and therapy response [4]. A mammogram is widely used for the early detection of breast cancer. However, other biomarkers are sought for the early detection of breast cancer, for example, in blood, nipple aspirate fluid, urine, or tears [6].

Furthermore, the following play crucial roles in prognosis and personalized treatment: genomic and immunohistochemical parameters; ER (estrogen receptor), PR (progesterone receptor), HER2, and Ki67(antigen Kiel 67) proliferation indices; and multigene panels. These biomarkers may influence therapeutic decisions and management strategies. Unfortunately, their reliability is limited due to interindividual variability [1].

According to the literature, we currently lack both prognostic biomarkers and therapeutic targets.

The most important prognostic factors are disease stage and tumor grade, but their roles have decreased over time. Most studies agree on the long-term-survival prognostic value of the mitotic index (MI), lymphovascular invasion (LVI), HER2 positivity, and gene profiling [7]. Comprehensive gene expression profiling has identified four major molecular subtypes of breast cancer, namely, luminal A breast cancer, luminal B breast cancer, HER2-positive breast cancer, and triple-negative breast cancer (TNBC), with TNBC accounting for 15–20% of all BCs. The subclassification of luminal A-like and luminal B-like breast cancers via immunohistochemistry (IHC) does not completely overlap the molecular subtypes because of BC heterogeneity, but it is currently used in clinical practice. They represent the most common subtypes of breast cancers, accounting for approximately 70% of cases [3,5,8,9]. The American Society of Clinical Oncology and the College of American Pathologists Guideline Recommendations state that the cutoff for positive ER or PR should be ≥1% of immunoreactive tumor cell nuclei (the previous threshold was >10%) [10].

Micro ribonucleic acids (miRNAs) are a group of endogenous non-coding RNAs that modulate gene expression at the post-transcriptional level and regulate diverse biological processes, such as proliferation, differentiation, migration, and apoptosis, acting as tumor suppressor genes or oncogenes. They can be significantly associated with TNBC prognosis [3,5].

Researchers still use histologic grade for breast cancer diagnosis, which is standard and associated with prognostic outcomes. Interobserver variability is the current challenge. Using artificial intelligence based on imaging features extracted from the sample region of interest can improve outcomes. Multiparametric feature sets containing imaging-based features and clinical characteristics have demonstrated high classification performance. In clinical datasets, clinicopathological characteristics such as Nottingham grades G1, 2, and 3 and pathologic biomarkers such as estrogen receptors, progesterone receptors, and human epidermal growth factor 2 are included [11,12].

Among the breast cancer subtypes, triple-negative breast cancer (TNBC) comprises a heterogeneous group of tumors with aggressive behavior and poor outcomes, and it represents 10–15% of all breast cancers. TNBC is characterized by a lack of expression of ER, PR, and HER2 receptors. Chemotherapy is the main therapeutic management for this type of breast cancer. New biomarkers have been identified, such as poly ADP-ribose polymerase (PARP) inhibitors, which have proven to be effective in the breast cancer genes BRCA1/2 subgroup of patients. Different gene expressions may aid in developing targeted and personal therapies, but only a few of these have shown an improvement in triple-negative breast cancer (TNCB) outcomes. In this regard, prognostics are needed in TNCB for clinical progress [13].

TILs (tumor infiltrating lymphocytes) are important biomarkers in TNCB and HER2-positive breast cancer, and they can be incorporated into clinical practice. A discordant TIL assessment is given by heterogeneity in the lymphocyte distribution, as well as additional factors such as technical slide-related issues, other inflammatory cells, and scoring variance between pathologists. TILs play a robust prognostic role [14]. Data suggest that, in TNCB patients, TILs are associated with the response to immunotherapy and cytotoxic treatments [15].

With stromal tumor infiltrating lymphocytes (TILs) being a prognostic biomarker in non-luminal breast cancer, researchers have evaluated their clinical relevance in luminal (hormone receptor-positive and HER2-negative) early breast cancer. Furthermore, TILs are associated with a better response to neoadjuvant chemotherapy but a poorer prognosis in luminal B cancer, higher proliferation, and a higher stage. Luminal BC is associated with less immune activation, and TILs’ significance is more uncertain. sTILs have been associated with higher biological and clinical aggressiveness, as well as tumor and lymph node proliferation and stage, among others. sTILs have been found to be predictive of pathologic complete response in patients treated with neoadjuvant chemotherapy [16].

It is unknown whether TILs play an immunological role in the different molecular subtypes of BC, and this issue remains controversial [16]. Studies have shown that high TILs are associated with statistically significantly better DFS and OS in patients with the HER2-positive molecular subtype and TNBC [17,18,19].

Ki67 is a non-histone nuclear protein present in the cell nucleus during all active phases of the cell, which makes it a widely used biomarker of tumor proliferation. Ki67 was established as a vital factor in the distinction between luminal A and luminal B breast cancer subtypes at the St Gallen International Breast Cancer Conference. However, the prognostic value is still unclear [10]. The 14% cutoff for the classification of luminal A and luminal B cancers was also proposed at the St Gallen International Breast Cancer Conference 2011 [10]. Antigen Ki67 is encoded by the MKI67 gene (marker of proliferation gene 67). The quantity of Ki67 is regulated by a precise balance between synthesis and degradation. It remains active through the G1, s, G2, and m phases of the cell cycle, making it an excellent marker of cell proliferation. The reliable prognostic value of Ki67 is now recognized in a number of cancers, including prostate, cervical, breast, and lung cancers. In clinical management, it is a reliable indicator of response to systemic therapy. There are difficulties in standardizing detection methods. Additionally, the misinterpretation of scoring may lead to inconsistencies in Ki67 reporting because of interpersonal variability. The current guidelines regarding the use of Ki67 in decision-making are controversial. Future digital image analyses will increase Ki67 utilization as a molecular biomarker for breast cancer [1]. Researchers established the Ki67 cutoff value that best predicts the benefit of chemotherapy. Core biopsies were stained for Ki67, and the findings were compared to the residual burden following neoadjuvant chemotherapy. An association between molecular subtype and pathological complete response was confirmed, and TILs had no effect on therapy response [20].

Researchers attempted to define predictive factors for different patterns of residual disease and compare the outcomes between scattered versus circumscribed patterns. Additionally, they emphasized that the discontinuation of neoadjuvant chemotherapy (NAC) cycles and tumor size are independent factors associated with patterns of residual disease. Scattered patterns showed better survival. The relationship between NAC, residual patterns, and differences in overall survival offers the potential to optimize therapeutic strategies [21].

The residual cancer burden (RCB) was also evaluated and found to correspond to better prognostic performance. TILs improved the performance of prognosis scores [22]. To avoid unnecessary neoadjuvant chemotherapy when anticipating a poor therapy response, it is essential to identify the pathological features that predict pathological complete response or at least a decrease in tumor burden following neoadjuvant chemotherapy [20].

The aim of this study was to evaluate biomarkers in the field of molecular pathology, along with classic biomarkers and their prognostic roles, to see their relevance and the need for new management, diagnostic, and therapeutic strategies.

## 2. Materials and Methods

We conducted an assessment of neoadjuvant therapy response with the aim of enhancing overall survival. This involved the radiographic, clinical, and pathological monitoring of tumor response prior to definitive surgery, enabling the subsequent evaluation of pathological response within a relatively short timeframe. Notably, the pathological evaluation of surgical specimens is widely regarded as the most accurate method for assessing chemotherapy efficacy.

### 2.1. Study Design

We conducted a retrospective analysis involving patients with early breast cancer or breast cancer who underwent neoadjuvant chemotherapy and/or endocrine therapy, immunotherapy, or radiotherapy at the Oncology Division of the Emergency County Hospital of Targu Mureș, Romania, before proceeding to curative surgical treatment. From a pool of 273 patients who underwent surgery at various surgery departments following neoadjuvant therapy between 1 January 2014, and 31 December 2023, we selected a subset for analysis. Patients without available follow-up data were excluded. Pathological procedures were conducted at the Department of Pathology, Emergency County Hospital of Targu Mureș, Romania, including the prospective assessment of the residual cancer burden (RCB) score and class, estrogen and progesterone receptor status, HER2 expression, and Ki67 labeling index. We retrospectively gathered clinicopathological data from biopsy reports and follow-up information from the local healthcare database, paper records, and telephone interviews.

For the assessment of ER and PR, the Allred score was used, and for the assessment of HER2, the 2013 ASCO/CAP (American Society of Clinical Oncology/College of American Pathologists) guidelines were used [23,24]. Ki67 was assessed via estimation using 10% steps, except for the area between 10% and 20%, for which 5% steps were used. If distinction between 10% steps was not possible, a range for Ki67 was included in the pathological report. Immunohistochemistry and in situ hybridization were performed on an automated stainer, a BenchMarkUltra TM, Roche-Ventana. Histological typing and grading were performed according to the WHO (World Health Organizatio) [25]. Carcinomas were categorized into molecular subgroups based on four immunohistochemical analyses. When luminal tumors were diagnosed, only select tumors with a higher stage or luminal B-like tumors with higher proliferation were selected for neoadjuvant chemotherapy.

In 2023, after a 10-year follow-up, survival data were updated, and 28.93% of patients received hormonotherapy, 67.39% received chemotherapy, 18.68% received Herceptin, 2.56% received radiotherapy, and 11.72% received no treatment at all being in the early stage of the disease. An MTA-1 Manual Tissue Arrayer was used (Beecher Instruments, Inc., Sun Prairie, WI, USA). Immunohistochemistry (IHC) for ER, PR, Ki67, and HER2 FISH was applied. Tumors were characterized histologically using WHO criteria [25], graded using the Nottingham system [9], and classified for subtype according to the following definitions: Luminal A (LA) means ER-positive (>=1%), PR > 20%, HER2 -negative, and Ki67 < 14%.

Luminal B (LB) means ER-positive, PR of more than 20%, and/or HER2 + and/or Ki67 of more than 14%.

HER2 enriched means ER of less than 1% with HER2 amplified;

Triple-negative breast cancer (TNBC) means ER of less than 1%, PR of less than 1 < 1%, and HER2-negative.

TILs were scored from HE sections of TMAs according to the published guidelines. Allred scores are based on ER proportion and based on immunohistochemical microscopy, the H-sore is based on staining nuclei, and cancer cellularity is based on a calculator of cellularity percentage from the HE section [25].

The RCB score was assessed using the RCB calculator on the MD Anderson website.

Informed consent was obtained from all subjects involved in the study, and the research was conducted in accordance with the Helsinki Declaration, with the approval of the Ethics Committee of Research UMFST Targu Mures for publication (No. 8656/31 May 2024).

### 2.2. Statistical Analyses

The Statistical Package for Social Sciences (SPSS, version 22, Chicago, IL, USA) was used to perform a statistical analysis. Nominal variables were characterized by means of frequencies.

The Kolmogorov–Smirnov test was used to analyze the quantitative variable distribution changes, which are described by mean ± standard deviation or median and percentiles (25; 75%) where appropriate. The frequencies of nominal variables were compared with a chi-square test. Differences in the mean between groups were analyzed using the *t* test or ANOVA where appropriate. Statistical significance was established at *p* < 0.05.

For quantitative variables with a Gaussian distribution, Student’s *t* test was applied, and for those without a Gaussian distribution, the Mann–Whitney test was applied. The chi-square test was used to assess the independence or association between variables. The Pearson coefficient was used to measure linear correlations between sets of data.

## 3. Results

Among the 273 patients, only 1 was male; the age varied from 23 to 90 years old, with a mean of 62.59.

We identified a statistically significant relationship between Nottingham grade and Ki67 (*p* = 0.0001): Grade I is associated with Ki67 less than 14, whereas grades II and III are associated with Ki67 over 14. The relationship between tumor grade and luminal is similar (*p* = 0.0001): Grade I is associated with luminal A, whereas grades II and III are associated with luminal B (Table 1).

Grade I seems to be statistically significantly corelated with TILs less than 10, whereas grades II and III are associated with TILs higher than 10 (*p* = 0.032). Regarding TNM stage, it is statistically significantly correlated with TILs (*p* = 0.01) and RCB (*p* = 0.0001). The early stage (in situ) is associated with TILs lower than 10, whereas TILs higher than 10 are found in the other stages. Regarding RCB, in situ stage correlates with RCB 0, whereas stages III and IV are associated with a high residual cancer burden and poor prognosis (Table 2).

**Figure 1:**
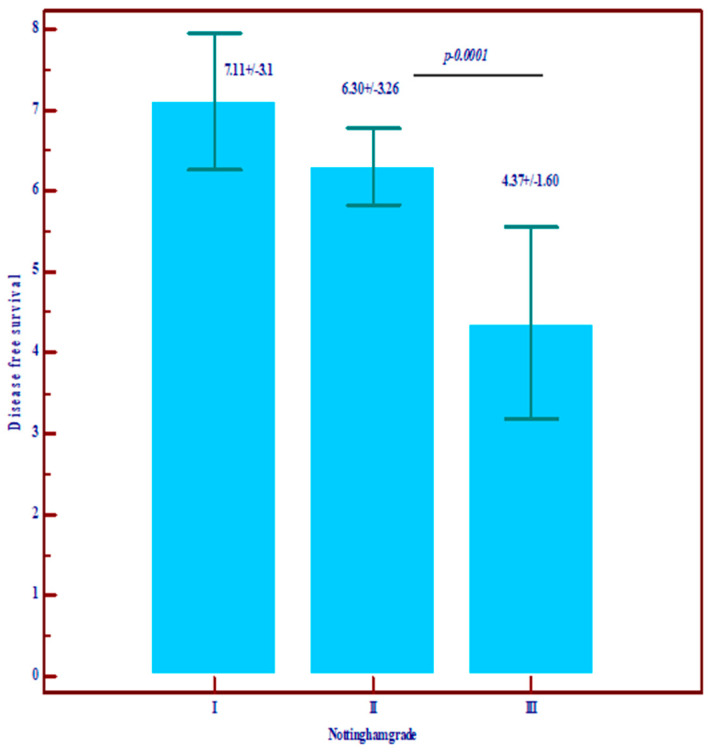

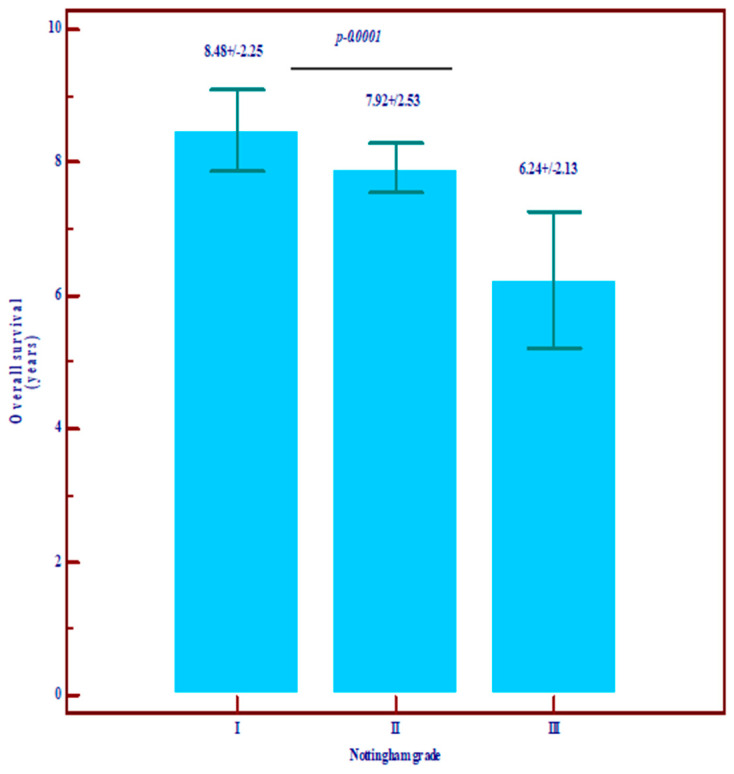
The relationship between Nottingham grade and overall survival (OS) and disease-free survival (data are expressed as mean and standard deviation).

**Figure 2:**
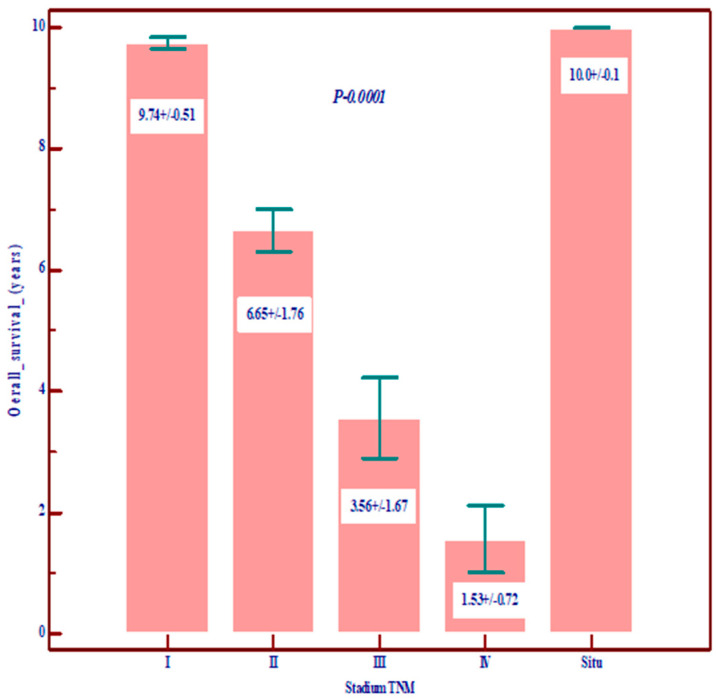

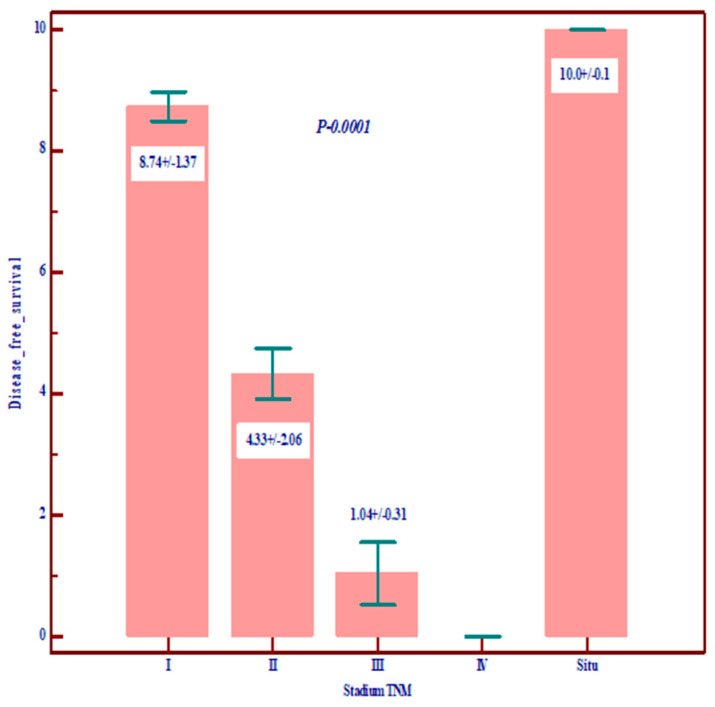
The relationship between TNM stage and overall survival (OS) and disease-free survival (data are expressed as mean and standard deviation).

**Figure 3:**
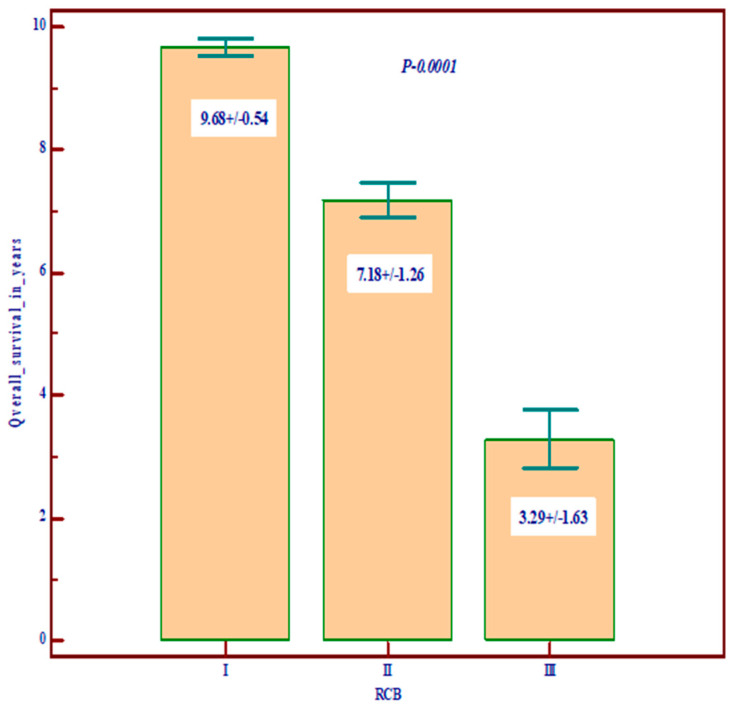

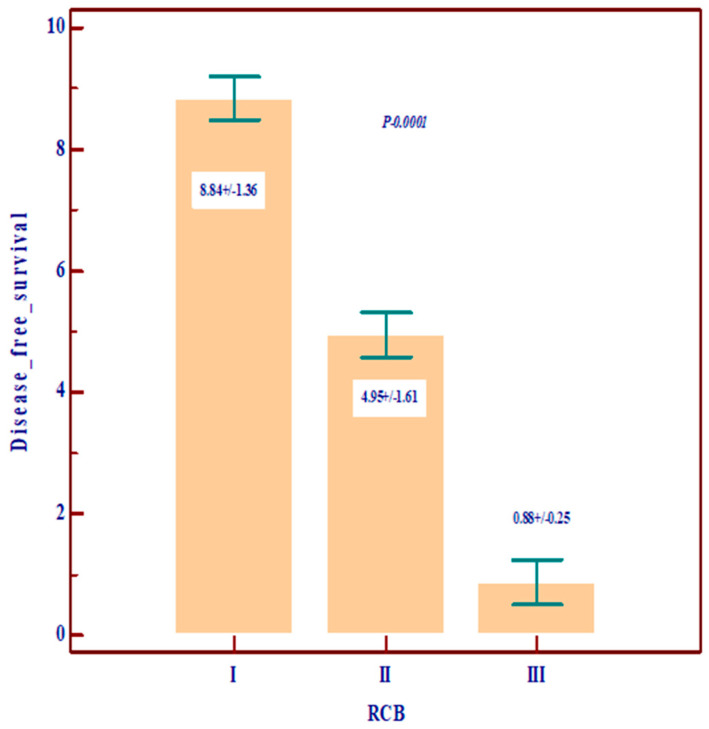
The relationship between RSB and overall survival (OS) and disease-free survival (data are expressed as mean and standard deviation).

Regarding the prognostic value, we noted that Nottingham grade 3 and TNM stages III and IV are correlated with low overall survival (OS) and disease-free survival (DSF), with a poor prognosis, and, among the molecular variables, the most important prognostic role is played by residual cancer burden (RCB) (Figure 1, Figure 2 and Figure 3).

Lymph node invasion is included both in tumor stage and in the RCB calculation, so we consider it a determining prognostic factor.

**Figure 4:**
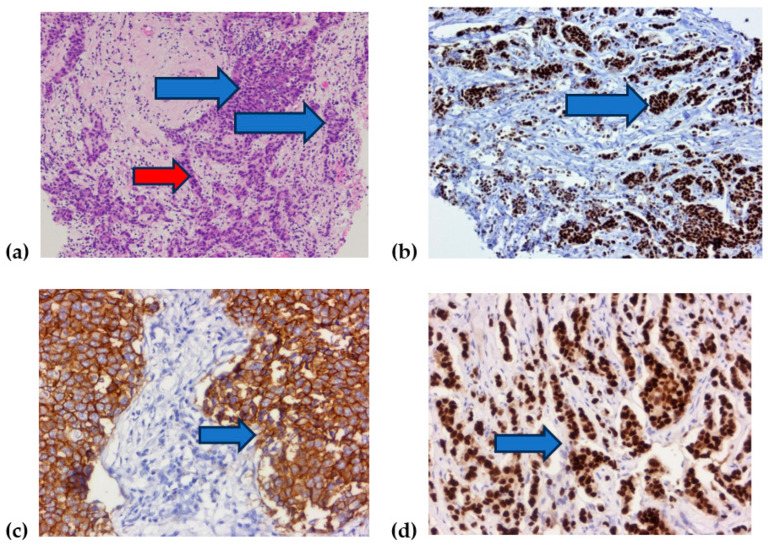
Histological images of breast cancer: (**a**) infiltrative ductal breast carcinoma placards (blue arrows) and trabecules (red arrow), (**b**) immunohistochemical reaction for estrogenic receptors (blue arrow), (**c**) immunohistochemical reaction for HER2 (blue arrow), (**d**) immunohistochemical reaction for progesteron receptor (blue arrow).

For better understanding of prognostic biomarkers compared in our retrospective study, we present in Figure 4 some examples of hematoxilin–eosin reaction microscopy and immunohistochemical reaction with some examples of molecular receptors used in luminal, H, and Allred scores (Figure 4).

The correlation between the neoadjuvant treatment used in patient management and the prognostic factors RCB and TNM, as well as how they influenced the prognosis, with statistical relevance, are shown in the tables below (Table 3 and Table 4).

As can be seen from Table 3 and Table 4, the treatment that correlates statistically significantly is chemotherapy with RCB and with TNM tumor stage. But in a percentage of 5.7%, despite the positive estrogen, progesterone, or HER2 receptors, the patients did not receive hormonal or immunotherapy, with the choice belonging to the patients. But this percentage does not influence the tables above.

The classic prognostic factors remain the reference, but molecular biological markers correlate with them. New targeted therapies will influence these results in the future.

## 4. Discussion

Traditional chemotherapy without a biomarker profile has been the main therapeutic management to date. This was also the main approach in our research. It was used in combination with hormone therapy or immunotherapy, depending on the presence of specific receptors, estrogen, progesterone, or HER2. Sometimes, radiotherapy was applied. In the case of early-stage disease, no preoperative treatment was used. In a small percentage of patients, that is, 5%, complementary therapy was not used, even if molecular biomarkers were determined, due to a lack of interdisciplinary communication. Such healthcare issues in Romania arise from insufficient funding and a shortage in medical personnel. Essential transformations are needed in our healthcare system [26].

Studies in the literature show that some patients receive neoadjuvant chemotherapy (NACT) without a clear benefit, being exposed to potential treatment-associated toxicity, and more effective therapeutic strategies are delayed. Thus, it is absolutely necessary to identify reliable predictive biomarkers [27]. According to our study, Ki67 of less than 14 is associated with luminal A, and a lower Nottingham grade is associated with a good prognosis.

Different biomarkers are sought in order to discover an effective therapeutic approach. Researchers still use Nottingham grade and tumor stage as prognostic factors, which are classic parameters; clinical datasets and molecular biology biomarkers such as estrogen receptors, progesterone receptors, and human epidermal growth factor 2 are recognized as useful tools in breast cancer progosis [11,12].

As emphasized in the introduction, the subclassification of luminal A-like and luminal B-like breast cancers via immunohistochemistry (IHC) does not completely overlap the molecular subtypes because of BC heterogeneity, even if it is currently used in clinical practice. The pCR rate for luminal B-like BC remains low, even with the modern regimens of neoadjuvant chemotherapy. New management strategies are sought [5]. The role of immunotherapy in hormone receptor (HR)-positive and HER2-negative BC remains unexplored. Cases with high TILs and PD-L1 expression appear to be more immune active [19].

The increased heterogeneity of BC complicates the management of this pathology in terms of diagnosis, prognosis, and treatment. For this reason, in their studies, the researchers sought to validate a simple pathology-based model to help clinicians identify BC subtypes in the absence of gene expression data. Molecular pathology plays an increasing role, but no validated biomarkers exist to identify those entities [28]. Residual cancer burden plays an important role in directly observing the effect of neoadjuvant therapy [29,30].

Breast tumors are considered a non-immunogenic disease, except for TNCB. The presence of TILs in TNCB plays predictive and prognostic roles. Increased TILs at diagnosis are associated with more favorable survival outcomes [13]. According to our study, high TILs are associated with a high Nottingham grade and a high TNM stage.

To avoid unnecessary neoadjuvant chemotherapy when anticipating a poor therapy response, it is essential to identify pathological features that predict pathological complete response or at least a decrease in tumor burden following neoadjuvant chemotherapy. TILs have no effect on therapy response [20]. TILs have a strong prognostic value in TNCB and HER2-enriched BC, but their prognostic role in luminal breast cancer is unclear. According to Zhang et al.’s study (2021), TILs are associated with more aggressive tumor features, but they do not appear to be associated with clinical outcome [31].

Other studies have shown that higher levels of TILs after systemic therapy are independent predictors of longer patient survival. Increased TIL was associated with chemoresponsiveness. Intratumoral TIL increased after therapy, and stromal TIL decreased in chemosensitive tumors; however, in chemoresistant patients, intratumoral TIL showed no significant change. These findings provided prognostic information for chemoresistant groups [32]. These aspects were not identified in our study. However, high TIL expression was not associated with the luminal molecular subtype of BC; conversely, high TILs were significantly associated with poor OS in patients with the luminal molecular subtype [17,18,19].

The limitations of this study are its single-center design; the limited number of patients, with missing data on complete pathological response; the way in which the recurrence of the disease and metastases were evaluated; and the anamnesis of disease-free survival being carried out by telephone. The strengths of this study lie in the relatively complex evaluation of prognostic and diagnostic molecular factors alongside classical ones, and the elimination of the variability in the anatomopathological evaluation, as only one pathologist performed the procedure.

## 5. Future Directions

Other immunological predictors of BC are sought. Inflammatory blood markers such as the neutrophil-to-lymphocyte ratio (NLR) have emerged as potential prognostic factors or predictive factors for pathological complete response (PCR) and toxicity in early or advanced BC. Most articles failed to find a significant correlation between NLR and PCR after neoadjuvant chemotherapy [33].

Considering that neoadjuvant chemotherapy is not the right solution in all situations, other approaches are sought. In this regard, scientists have discovered that BRCA1/2 (breast cancer types 1 and 2) are involved in DNA repair. These findings led to the development of new therapies, such as poly ADP-ribose polymerase (PARP) inhibitors [13].

Furthermore, PD-L1 is an immune checkpoint and represents an adaptative immune mechanism that helps cancer cells escape. PD-1/PD-L1 ligation acts as a pro-tumorigenic pathway and is expressed in TNBC [13,19]. These promising molecular biomarkers can lead to new therapeutic approaches, and their detection in blood or other fluids can facilitate prognosis or the monitoring of the response to drugs [13].

Molecular tools and multigene signatures help with making decisions on the extension of chemotherapy and hormone therapy, especially in patients with early-stage disease for whom toxic effects are particularly deleterious or this treatment is not necessary. Such predictive biomarkers helps us avoid the toxicity of standard chemotherapy and evaluate the eligibility of patients for targeted therapy. The Cancer Genome Atlas project has created the possibility of comparing whole genomes and helps us identify novel biomarkers [34,35].

Personalized oncology is based on genomics, genetic differences in individuals, and the variability in the clinical response to chemotherapy. Germline mutations and DNA methylation can lead to the modification of gene expression, and elevated levels of miRNA capable of binding messenger RNA affect gene expression, with a poor prognosis for the patient. This type of molecular diagnostic is used in chronic myeloid leukemia, colon cancer, and lung cancer, with benefits in targeted treatment. It can also be applied to BC [36]. Long non-coding RNAs play a crucial role in the stemness regulation of breast cancer stem cells. They have prognostic value in breast cancer, with promising effectiveness in clinical practice [37].

Multigene signatures are mainly used when clinical parameters and traditional immunohistochemical biomarkers alone lead to equivocal prognosis. Molecular tools help with making decisions on the extension of adjuvant endocrine or chemotherapy or when this treatment is not needed. This approach is mandatory in very heterogeneous carcinomas and may improve the clinical management of the disease [34]. Online tools are able to identify gene expression-based biomarkers using transcriptomic data [4].

There is a need for an innovative approach to identify effective tools in the management of BC. Phosphatase and tensin homolog deleted on chromosome 10 (PTEN) is a tumor suppressor gene that regulates cell proliferation, cell mobility, enzymatic or non-enzymatic activities, and phosphatidylinositol 3-kinase PI3K-dependent and -independent mechanisms. However, there is no clinically validated detection assay that can be used to analyze PTEN expression or function [38].

Other treatments are also sought for when neoadjuvant chemotherapy proves to be toxic—for example, genistein, which is a flavonoid compound with antiproliferative, antiangiogenic, and anti-inflammatory properties [39]. Moreover, reproductive changes have to be taken into account as factors that could influence breast cancer evolution [40].

Traditional prognostic factors persist, and more recent factors need further follow-up [7]. But RCB should become a standard pathology evaluation after neoadjuvant therapy [41,42].

## 6. Conclusions

Molecular tools help decide the appropriate neoadjuvant therapy. These, together with classic prognostic factors, help avoid chemotherapy toxicity, especially in the early stages of the disease, when personalized therapy can be used. Our research also emphasizes the importance of traditional prognostic factors while underlining the role of Ki67, which is positively correlated with the Nottingham grade, as well as with the luminal subtypes. TILs are positively correlated with grade and stage. Residual cancer burden seems to have significant importance as a prognostic factor. However, these factors need further validation. Multidisciplinarity in breast cancer management is of major importance.

## Figures and Tables

**Table 1:** The relationship between molecular variables and Nottingham grade.

Variables		Nottingham Grade			Total	*p*-Value
		I	II	III		
Ki67 (1 means < 14 and has good prognosis)	1	28 (51.9)	49 (27.1)	8 (21.1)	85 (31.1)	0.0001 Statistically significant
	2	26 (48.1)	132 (72.9)	30 (78.9)	188 (68.9)	
HER	0	39 (72.2)	107 (59.1)	23 (60.5)	169 (61.9)	*p* = 0.10
	1	11 (20.4)	38 (21.0)	4 (10.5)	53 (19.4)	
	2	0 (0.0)	13 (7.2)	5 (13.2)	18 (6.6)	
	3	4 (7.4)	23 (12.7)	6 (15.8)	33 (12.1)	
Luminal category	A	22 (40.7)	39 (21.5)	4 (10.5)	65 (23.8)	*p* = 0.0001 Statistically significant
	B	13 (24.1)	59 (32.6)	13 (34.2)	85 (31.1)	
	B (HER2)	12 (22.2)	59 (32.6)	5 (13.2)	76 (27.8)	
	HER2	2 (3.7)	4 (2.2)	8 (21.1)	14 (5.1)	
	TNBC	5 (9.3)	20 (11.0)	8 (21.2)	33 (12.1)	
TILs (1 means low prognosis)	1	25 (46.3)	50 (27.6)	13 (34.2)	88 (32.2)	*p* = 0.032 Statistically significant
	2	29 (53.7)	131 (72.4)	25 (65.8)	185 (67.8)	
RCB	0	17 (31.5)	62 (34.3)	9 (23.7)	88 (32.2)	*p* = 0.10
	1	17 (31.5)	34 (18.8)	6 (15.8)	57 (20.9)	
	2	14 (25.9)	54 (29.8)	11 (28.9)	79 (28.9)	
	3	6 (11.1)	31 (17.1)	12 (31.6)	49 (17.9)	
R estrogen H Score	Mean ± SD	232.78 ± 73.4	221.83 ± 80.3	168.0 ± 105.5	218.8 ± 83.2	Statistically significantly lower values in grade III (*p* = 0.008)
R estrogen Allred score	Mean ± SD	7.43 ± 1.25	7.34 ± 1.47	6.23 ± 2.13	7.25 ± 1.53	Statistically significantly lower values in grade III (*p* = 0.007)
R progesterone H score	Mean ± SD	163.2 ± 37.4	148.4 ± 38.16	90.32 ± 32.58	146.23 ± 38.84	Statistically significantly lower values in grade III (*p* = 0.021)
R progesterone Allred sScore	Mean ± SD	6.20 ± 1.94	6.30 ± 2.01	4.42 ± 2.21	6.11 ± 2.07	Statistically significantly lower values in grade III (*p* = 0.004)
Overall survival in years	Mean ± SD	8.48 ± 2.25	7.92 ± 2.53	6.24 ± 2.13	7.79 ± 2.65	Statistically significantly lower values in grade III (*p =* 0.0001)
Disease-free survival (DFS)	Mean ± SD	7.11 ± 3.10	6.30 ± 3.26	4.37 ± 1.60	3.19 ± 3.36	Statistically significantly lower values in grade III (*p* = 0.0001)

**Table 2:** Correlations of molecular variables, TNM.

Variables		Stadium TNM					Total	*p*-Value
		I	II	III	In Situ	IV		
Ki67 (1 means < 14 and has good prognosis)	1	49 (36.8)	25 (26.3)	7 (25.9)	3 (33.3)	1 (11.1)	85 (31.1)	*p* = 0.28
	2	84 (63.2)	70 (73.7)	20 (74.1)	6 (66.7)	8 (88.9)	188 (68.9)	
HER2	0	84 (63.2)	59 (62.1)	14 (51.9)	6 (66.7)	6 (66.7)	169 (61.9)	*p* = 0.36
	1	22 (16.5)	21 (22.1)	9 (33.3)	0 (0.0)	1 (11.1)	53 (19.4)	
	2	10 (7.5)	6 (6.3)	2 (7.4)	0 (0.0)	0 (0.0)	18 (6.6)	
	3	17 (12.8)	9 (9.5)	2 (7.4)	3 (33.3)	2 (22.2)	33 (12.1)	
Luminal category	A	37 (27.8)	20 (21.1)	5 (18.5)	2 (22.2)	1 (11.1)	65 (23.8)	*p* = 0.75
	B	40 (30.1)	32 (33.7)	6 (22.2)	2 (22.2)	5 (55.6)	85 (31.1)	
	B (HER2)	39 (29.3)	24 (25.3)	10 (37.0)	2 (22.2)	1 (11.1)	76 (27.8)	
	HER2	4 (3.0)	6 (6.3)	2 (7.4)	1 (11.1)	1 (11.1)	14 (5.1)	
	TNBC	13 (9.8)	13 (13.7)	4 (14.8)	2 (22.2)	1 (11.1)	33 (12.1)	
TILs (1 means low prognosis)	1	47 (35.3)	22 (23.2)	9 (33.3)	7 (77.8)	3 (33.3)	88 (32.2)	*p* = 0.01 Statistically significant
	2	86 (64.7)	73 (76.8)	18 (66.7)	2 (22.2)	6 (66.7)	185 (67.8)	
RCB	0	74 (55.6)	6 (6.3)	1 (3.7)	7 (77.8)	0 (0.0)	88 (32.2)	*p* = 0.0001 Statistically significant
	1	52 (39.1)	3 (3.2)	0 (0.0)	2 (22.2)	0 (0.0)	57 (20.9)	
	2	7 (5.3)	68 (71.6)	4 (14.8)	0 (0.0)	0 (0.0)	79 (28.9)	
	3	0 (0.0)	18 (18.9)	22 (81.5)	0 (0.0)	9 (100)	49 (17.9)	
R estrogen H score	Mean ± SD	217.63 ± 85.7	224.05 ± 75.3	217.6 ± 88.5	180.83 ± 98.92	218.57 ± 82.5	218.8 ± 83.2	Statistically insignificant
R estrogen Allred score	Mean ± SD	7.29 ± 1.48	7.27 ± 1.46	7.14 ± 1.83	6.33 ± 2.42	7.43 ± 1.51	7.25 ± 1.53	Statistically insignificant
R progesterone H score	Mean ± SD	144.45 ± 54.01	141.41 ± 35.26	137.11 ± 35.66	247.5 ± 59.1	188.57 ± 52.8	146.23 ± 38.8	Statistically insignificant
R progesterone Allred score	Mean ± SD	6.28 ± 1.92	5.83 ± 2.19	5.61 ± 2.38	7.75 ± 0.5	6.71 ± 2.36	6.11 ± 2.07	Statistically insignificant
Overall survival in years	Mean ± SD	9.74 ± 0.51	6.65 ± 1.76	3.56 ± 1.67	10.0 ± 0.1	1.56 ± 0.72	7.79 ± 2.65	0.0001 Statistically significant lower value in stages III and IV
Disease-free survival (DFS)	Mean ± SD	8.74 ± 1.37	4.33 ± 2.06	1.04 ± 0.31	10.0 ± 0.1	-	6.19 ± 2.36	0.0001 Statistically significant lower value in stages III and IV

**Table 3:** Neoadjuvant treatment in correlation with RCB.

			RCB			Total	
			I	II	III		*p* Value
Chemotherapy	yes	Count	36	65	43	144	0.0001
		%	63.2%	82.3%	87.8%	77.8%	
Hormontherapy	yes	Count	24	24	13	61	0.19
		%	42.1%	30.4%	26.5%	33.0%	
Herceptin	yes	Count	9	18	12	39	0.49
		%	15.8%	22.8%	24.5%	21.1%	
Radiotherapy	yes	Count	3	2	1	6	0.57
		%	5.3%	2.5%	2.0%	3.2%	

**Table 4:** Neoadjuvant treatment in correlation with TNM.

			Stadium TNM					Total	
			I	II	III	In Situ	IV		*p* Value
Chemotherapy	yes	Count	70	75	25	4	9	183	0.0001
		%	52.6%	78.9%	92.6%	44.4%	100.0%	67.0%	
Hormontherapy	yes	Count	41	28	10	0	0	79	0.072
		%	30.8%	29.5%	37.0%	0.0%	0.0%	28.9%	
Herceptin	yes	Count	22	21	5	3	2	53	0.70
		%	16.5%	22.1%	18.5%	33.3%	22.2%	19.4%	
Radiotherapy	yes	Count	5	2	1	0	0	8	0.93
		%	3.8%	2.1%	3.7%	0.0%	0.0%	2.9%	

## Data Availability

Data are unavailable due to ethical restriction.
